# Assessment of the measurement methods in midshaft clavicle fracture

**DOI:** 10.1186/s12891-022-05961-y

**Published:** 2022-11-18

**Authors:** Guilherme Vieira Lima, Vitor La Banca, Joel Murachovsky, Luis Gustavo Prata Nascimento, Luiz Henrique Oliveira Almeida, Roberto Yukio Ikemoto

**Affiliations:** 1 Grupo de Ombro e Cotovelo da Faculdade de Medicina do ABC, Av. Lauro Gomes, 2000 - Vila Sacadura Cabral, Santo André, SP 09060-870 Brazil; 2 Rua Estela 121, Apto 141, Paraíso, São Paulo, SP CEP: 04011-001 Brazil; 3 Departamento de Ortopedia do Hospital Ipiranga, Av. Nazaré, 28 - Vila Monumento, São Paulo, SP 04262-000 Brazil

**Keywords:** Clavicle, Bone fractures, Radiography, Methods

## Abstract

**Background:**

Clavicle fractures account for approximately 5% of all fractures in adults and 75% of clavicle fractures occur in the midshaft. Shortening greater than two centimeters is an indicative of surgical treatment. Radiographic exams are often used to diagnose and evaluate clavicle fractures but computed tomography (CT) scan is currently considered the best method to assess these deformities and shortening.

**Goal:**

1- To investigate whether different methods of performing the radiographic exam interfere on the measurement of the fractured clavicle length.

2- Compare the clavicle length measurements obtained by the different radiographic exam methods with the CT scan measurements, used as a reference.

**Materials and methods:**

Twenty-five patients with acute (< 3 weeks) midshaft clavicle fracture were evaluated. Patients underwent six radiographic images: PA Thorax (standing and lying), AP Thorax (standing and lying) and at 10° cephalic tilt (standing and lying), and the computed tomography was used as reference.

**Results:**

The mean length (cm) obtained were: 14,930 on CT scan, 14,860 on PA Thorax Standing, 14,955 on PA Thorax Lying, 14,896 on AP Thorax Standing, 14,960 AP Thorax Lying, 15,098 on 10° cephalic tilt Standing and 15,001 on 10° cephalic tilt Lying, (*p* > 0,05).

**Conclusion:**

1- There is no significant statistical difference in the clavicle fracture length measurement among the variety of radiographic exam performances.

2- The method that comes closest to computed tomography results is the PA thorax incidence, with the patient in the lying position.

## Background

Clavicle fractures account for approximately 5% of all fractures in adults [1] and 44% of all injuries around the shoulder girdle [2]. 75% of clavicle fractures occur in the midshaft [1–4] and non-surgical treatment of midshaft fractures has showed good results [1, 4, 5]. However, these results have been recently questioned by several authors who have shown worse outcomes in patients with clavicle fractures consolidated at a shortening of more than two centimeters [6–14]. They consider shortening greater than two centimeters an indicative of surgical treatment [10–14].

A clavicle fracture diagnosis can be easily performed by physical exam. However, an assessment with image exams is required in order to specify deviations, shortenings and its precise location [15]. Due to the multiplanar nature of this bone, it is hard to determine the angle deviation and spatial arrangement of the fragments through a single radiographic analysis [16–19]. Some authors suggest that four incidences lead to a better fracture evaluation, and computed tomography (CT) scan is currently considered the best method to assess these deformities [16, 18, 20–25].

The patient’s position during radiographic affects the image obtained by this exam [16, 26, 27]. Radiographies performed while the patient is in an orthostatic position show different angle deviations from those performed in a supine position, due to the action of the gravitational force [26, 27]. Moreover, the distance between the study object and the x-ray capture surface has an impact on the image magnification [16]. In radiographies performed in PA view (where the incident beams direction goes from posterior to anterior), the distance between the clavicle and the film is different from the one got in AP view (where the incident beams direction goes from anterior to posterior). Thus, the different ways of performing the exam impact on the values obtained in radiographic measurements [16, 18]. Therefore, this study aims to investigate whether different methods of performing the radiographic exam interfere with the measurement of the fractured clavicle length, and to compare the clavicle length measurements obtained by each type of radiographic exam with the measurement got from CT scan. In this study, we will also assess which radiographic method shows the most reliable measurement for a fractured clavicle length in comparison with the measurement obtained from CT (used as a reference).

## Materials and methods

Twenty-five patients with clavicle fracture were evaluated at our emergency service. Acute midshaft clavicle fractures that had progressed less than 3 weeks were included, showing with simple or comminuted traits, exhibiting deviations or not (2A1, 2A2, 2B1 and 2B2 types from Robinson). Patients attended were both sexes, ages ranging from 18 to 69 years old, who agreed to be part of this study (with informed consent). Proximal or distal third clavicle fractures, fractures that had progressed for more than 3 weeks, previous clavicle fractures and/or showing other fractures or associated shoulder girdle injuries were not included.

**Fig. 1:**
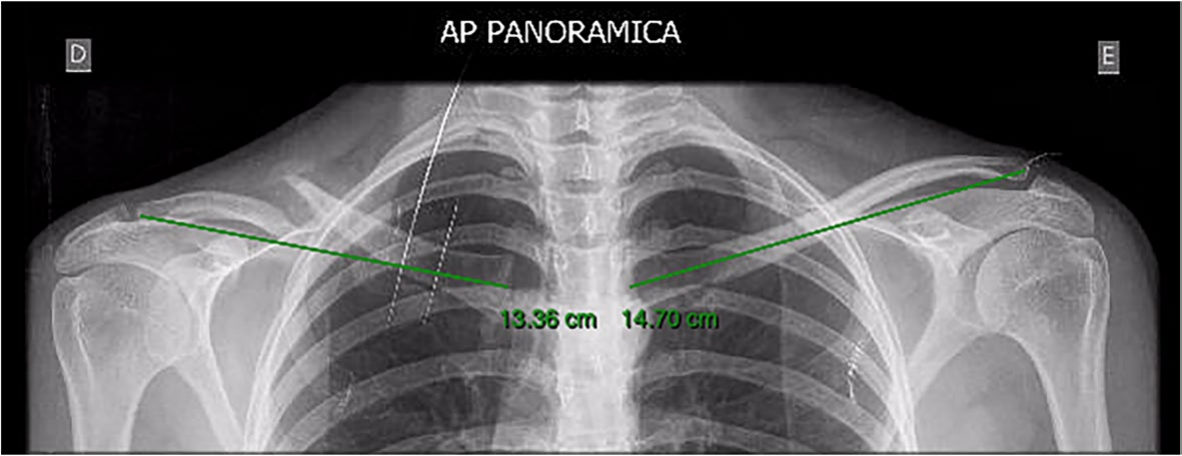
Clavicle length measurement technique

Age, sex, high, weight, trauma mechanism and affected side data were collected. Patients underwent six radiographic images: PA Thorax (standing and lying), AP Thorax (standing and lying) and at 10° cephalic tilt (standing and lying) [28]. All images were obtained in order to include both clavicles in their entirety (distal and proximal articulation). An image analysis program (Vue Motion, Carestream - Rochester, NY) was used to measure clavicle length by means of a technique described by Smekal et al. [16], and Lazaride et al. [25], connecting most medial to the most lateral point of the clavicle (Fig. 1).

**Fig. 2:**
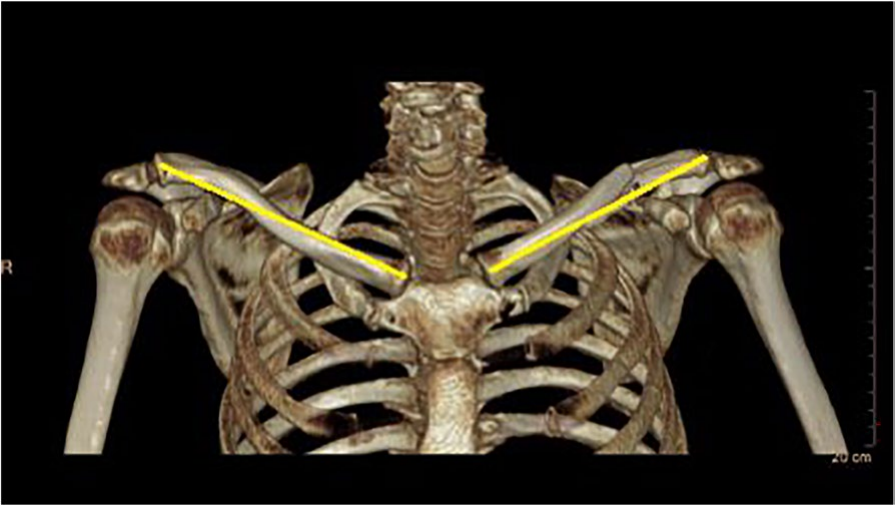
Measurement of clavicle length by computed tomography

The same length measurement method (connecting most medial to the most lateral point of the clavicle) was used in the 3D computed tomography obtained images as shown in Fig. 2. From the length measurement, the relative shortening for each patient was calculated, subtracting the fractured clavicle length value from the length of the integral clavicle.

Four of the 25 patients were excluded. Two presented contraindications to CT and two presented unsatisfactory tomographic images.

Dependent t-test was used for data with normal distribution and Wolcoxon test for those who didn’t show normal distribution. Significance level was 5%. The software used was version 11.0 Stata (STATACORP, LC).

The research ethics committee approved this study according to the Helsing protocol.

## Results

Of the 21 patients, 15 were male and 6 were female. Mean age was 41.2 (18–69) years old, mean height was 1.73 (1.54–1.91) meters and the mean weight was 72.8 (46–110) kilograms. The mechanism trauma in 16 cases were a fall directly on the shoulder with the arm at the side and 5 were indirect trauma from a fall on an outstretched hand (Table 1).

**Table 1:** Sample information

Sample	Age	Gender (M-Male / F-Female)	Weight (Kilogram)	High (meters)	Affected Side	Trauma Mechanism	Frature Type
1	34	M	100	1,82	Right	Direct	Simple
2	27	F	68	1,63	Right	Direct	Simple
3	35	M	86	1,78	Right	Direct	Complex
4	18	M	68	1,9	Left	Direct	Simple
5	22	F	56	1,62	Right	Direct	Complex
7	58	M	76	1,6	Left	Direct	Complex
8	53	M	53	1,81	Left	Direct	Complex
9	30	F	70	1,75	Right	Direct	Simple
10	35	M	83	1,84	Right	Direct	Simple
11	61	M	67	1,72	Left	Direct	Simple
12	61	M	66	1,79	Right	Direct	Simple
13	18	M	70	1,75	Right	Direct	Simple
14	57	M	86	1,76	Right	Direct	Simple
15	22	F	46	1,65	Left	Direct	Simple
16	25	M	89	1,74	Left	Direct	Complex
17	35	M	110	1,91	Left	Indirect	Simple
18	64	F	62	1,54	Left	Indirect	Complex
19	58	M	76	1,72	Right	Direct	Simple
20	26	M	75	1,76	Left	Indirect	Simple
21	69	M	62	1,63	Right	Indirect	Complex
22	58	M	60	1,64	Right	Indirect	Simple

Regarding the affected side, 12 patients had their fracture on the right side and 9 on the left. The dominant side was affected in 13 patients and the non-dominant in 8. According to Robinsons et al. [1] classification, 14 fractures were simple (2A1/2A2/2B1) and 7 were comminuted fractures (2B2).

There wasn’t any significant statistical difference in the clavicle fracture length measurement among the variety of radiographic exam performances when compared to CT (Table 2).

**Table 2:** Length Measurement of the Fractured Clavicle in Radiographic Exams and Tomography

Exam	Mean Length (cm)	Mean difference compared to CT (cm)	95% CI	***p***
**Tomography**	14,930 (14,378-15,481)	–	–	–
**PA Thorax Standing**	14,860 (14,252-15,4680)	0,698	(−0,134/0,274)	0,4848
**PA Thorax Lying**	14,955 (14,357 - 15,553)	−0,025	(− 0,197/0,147)	0,762
**AP Thorax Standing**	14,896 (14,351-15,441)	0,033	(−0,126/ 0,192)	0,6678
**AP Thorax Lying**	14,960 (14,385-15,535)	-0,03	(−0,196/0,136)	0,7096
**10° cephalic tilt Standing**	15,098 (14,513 - 15,682)	-0,168	(−0,400/0,063)	0,1456
**10° cephalic tilt Lying**	15,001 (14,422 - 15,580)	-0,071	(−0,235/0,092)	0,3742

The comparison between the obtained measurements with different patient positions (standing or lying) did not demonstrate any significant statistical difference (*p* = 0,376) (Table 3). Measurement values obtained by different X-ray incidences (AP or PA) were also similar (*p* = 0,732) (Table 4).

**Table 3:** Comparison between standing AP and lying AP

	Mean measurement cm	95% CI	
**AP Thorax Lying**	14,960 (14,385-15,535)	(14,385/15,535)	
**AP Thorax Standing**	14,896 (14,351-15,441)	(14,351/15,441)	
**Difference**	-0,063	(−0,209/0,082)	*p* = 0,3762

**Table 4:** Comparison between standing AP thorax and standing PA thorax

	Mean measurement cm	95% CI	
**Standing PA Thorax**	14,860 (14,252-15,468)	(14,252/15,4680)	
**Standing AP Thorax**	14,896 (14,351-15,441)	(14,351/15,441)	
**Difference**	0,036	(−0,183/0,256)	*p* = 0,732

In the shortening evaluation, significant differences weren’t observed between orthostatic standing and lying exams (*p* = 0,204), not even between AP and PA incidences (*p* = 0,531), considering the integral clavicle length as a parameter.

## Discussion

Clavicle shortening is considered one of the main parameters to make a surgical referral, however, there is no consensus on the exact value of this measure. Lazarides et al. [25] considered bad prognosis for the conservative treatment fractures with shortenings greater than 1,4 cm in women and 1,8 cm in men. Hill et al. [9], consider shortening values greater than 2,0 cm to indicate surgery and they don’t mention gender differences in their study. Postattine et al. [29] consider shortening as the fracture overlap percentage, being referred to surgery those who present a percentage of overlap greater than 13 to 15% of the total length. And De Giorgis et al. [30] parameter is a shortening percentage of 9,7% in comparison to the integral clavicle length.

Regarding the variation of the clavicle length among individuals, Daruwalla et al. [20] showed, through integral clavicles tomographic measurement, that there is a variation of 129.4 mm to 161.2 mm in that bone’s size, and King et al. [21] found a length variation of 121.5 to 183.3 mm. In our study, we observed a variation from 132 mm to 169 mm and, therefore, using an absolute but not relative percentage shortening value may cause different effects according to the clavicle size.

As well as Lazarides et al. [25], we measured the clavicle shortening from the contra lateral clavicle length measure, as a parameter of normal length. Some authors measure the shortening from the edges of the fractured fragments in a straight-line projection [16–18, 31]. That methodology may not be precise due to the “S” shape [11, 15] characteristic of that bone and to the comminution of many clavicle fractures, associated with images overlap of a simple radiography [23].

There is no consensus over X-ray tilt. We found, in our literature search, a wide angular variation in the incidence of these rays. Studies show exams performed at the following angles: 0°, 10° cephalic, 15° caudal, 20° caudal, 20° cephalic, 30° cephalic, 45° cephalic and 45° caudal [1–28, 31–33], in neither of which there is an explanation for this great angular variation. In our study, we standardized inclinations to 0° (due to its vast use by most authors) and 10° cephalic (for being the incidence that better evaluates the limits of the acromioclavicular articulation for a precise assessment of that bone’s lateral boundary).

Currently, CT exam is considered the gold standard method [16, 18, 20–25]. It allows a detailed assessment of the bone fragments, as well as a three-dimensional assessment through one simple exam. Omid et al. [24] show the superiority of a CT over a simple radiography in the evaluation of the fractured clavicle shortening. However, one of the main questions regarding the comparative radiography and tomography studies is in relation to the positioning of the patient. When lying down, the gravitational force vector relative to the patient is different from a standing position, and that could affect the measurement of the fractured clavicle length. Onizuka et al. [26] made a similar assessment of the gravitational impact, comparing only AP X-rays of the fractured clavicle with a 15° tilt performed with a patient standing and then lying. The authors obtained a significant difference in the angular measurement and vertical deviation, however, as we did in our study, they observed no change in length and shortening.

In this study, we searched for the radiographic incidence that presents the most reliable length measurements of the fractured clavicle in comparison with the measurements obtained in CT. Lying PA incidence was the one that showed values closest to the tomographic exam. Other studies make similar assessments [16, 18, 24], however, none of the studies in our survey included the AP and PA incidences in patients standing and then lying. Different from the results obtained by Smekel et al. [16], our study did not show a significant statistical difference between AP and PA incidences. Despite the lying PA exam being the most reliable CT to the clavicle length measurements, it was also the one in which patients complained of much pain during the exam. As there is no statistically significant difference between the exams, we do not recommend this incidence performance in order to avoid unnecessary discomfort to the patients.

We also did not assess the relation between fracture time and shortening pattern. In our evaluation, we included patients whose fractures had progressed less than 3 weeks, and according to Onizuka et al. [26], it is in this interval most deviations occur. After 21 days, the presence of fibrous scar tissue stabilizes the fracture and prevents the displacement of fragments even when the patient changes position.

The main limitation of our study was the sample size, nevertheless the statistic showed that there is no difference between the described methods but futures studies with a larger number are necessary. Other limitations were: heterogeneous distribution between male and female individuals, we did not consider spinal and postural disorders, nor the biotype of individuals that can affect the clavicle positioning during a radiography exam. Despite the radiographic technique being standardized in relation to the patient positioning during examination, the execution of a good quality exam depends on experienced technicians. In our study, we had to repeat the radiographic exam in three patients because of unsatisfactory exams that did not include both clavicles in their entirety.

The shortening of the fractured clavicle is one of the main parameters for surgical indication. Since there is no statistical difference between the methods presented, the study showed us that it is possible to evaluate the length of the fractured clavicle using any of the radiographic methods described.

If there was a significant statistical difference in our study, we would be able to standardize a single radiographic method to the assessment of the fractured clavicle length and facilitate conduct taking.

## Conclusion

Our results show that there is no significant statistical difference in the clavicle fracture length measurement among the variety of radiographic exam performances made in this study, and the method that comes closest to computed tomography results is the PA thorax incidence, with the patient in the lying position. However, it is not recommended due to the intensification of pain.

## Data Availability

The datasets generated and/or analyzed during the current study are not publicly available due to limitations of ethical approval involving the patient data and anonymity but are available from the corresponding author on reasonable request.

## Abbreviations

CT: Computed tomography
PA: Posterior anterior
AP: Anterior posterior
